# Local CD34-positive capillaries decrease in mouse models of kidney disease associating with the severity of glomerular and tubulointerstitial lesions

**DOI:** 10.1186/s12882-017-0694-3

**Published:** 2017-09-04

**Authors:** Md Abdul Masum, Osamu Ichii, Yaser Hosny Ali Elewa, Teppei Nakamura, Yasuhiro Kon

**Affiliations:** 1 Laboratory of Anatomy, Graduate School of Veterinary Medicine, Hokkaido University, Kita 18, Nishi 9, Kita-ku, Sapporo, Japan; 2 Department of Histology, Faculty of Veterinary Medicine, Zagazig University, Zagazig, Egypt; 3 Section of Biological Safety Research, Chitose Laboratory, Japan Food Research Laboratories, Tokyo, Japan

**Keywords:** CD34, Capillary, Kidney disease, Glomerular lesion, Tubulointerstitial lesion

## Abstract

**Background:**

The renal vasculature plays important roles in both homeostasis and pathology. In this study, we examined pathological changes in the renal microvascular in mouse models of kidney diseases.

**Methods:**

Glomerular lesions (GLs) in autoimmune disease-prone male BXSB/MpJ-*Yaa* (Yaa) mice and tubulointerstitial lesions (TILs) in male C57BL/6 mice subjected to unilateral ureteral obstruction (UUO) for 7 days were studied. Collected kidneys were examined using histopathological techniques. A nonparametric Mann–Whitney *U* test (*P* < 0.05) was performed to compare healthy controls and the experimental mice. The Kruskal-Wallis test was used to compare three or more groups, and multiple comparisons were performed using Scheffe’s method when significant differences were observed (*P* < 0.05).

**Results:**

Yaa mice developed severe autoimmune glomerulonephritis, and the number of CD34^+^ glomerular capillaries decreased significantly in GLs compared to that in control mice. However, UUO-treated mice showed severe TILs only, and CD34^+^ tubulointerstitial capillaries were decreased significantly in TILs with the progression of tubulointerstitial fibrosis compared to those in untreated control kidneys. Infiltrations of B-cells, T-cells, and macrophages increased significantly in the respective lesions of both disease models (*P* < 0.05). In observations of vascular corrosion casts by scanning electron microscopy and of microfil rubber-perfused thick kidney sections by fluorescence microscopy, segmental absences of capillaries were observed in the GLs and TILs of Yaa and UUO-treated mice, respectively. Further, transmission electron microscopy revealed capillary endothelial injury in the respective lesions of both models. The numbers of CD34^+^ glomerular and tubulointerstitial capillaries were negatively correlated with all examined parameters in GLs (*P* < 0.05) and TILs (*P* < 0.01), respectively.

**Conclusions:**

From the analysis of mouse models, we identified inverse pathological correlations between the number of local capillaries in GLs and TILs and the severity of kidney diseases.

**Electronic supplementary material:**

The online version of this article (10.1186/s12882-017-0694-3) contains supplementary material, which is available to authorized users.

## Background

Kidney disease causes a systemic deterioration in health. Chronic kidney disease, in particular, is a substantial public health burden because it is associated with end stage renal disease and cardiovascular disease [1]. Various intrinsic and extrinsic factors, such as genomic factors, infections, and drug use, affect the progression of kidney diseases. The kidney is a highly vascularized organ, renal vasculature is also believed to play important roles in both renal function and tissue homeostasis. Altered vasculature can cause several renal injuries mediated by hypoxia-associated processes [2–4]. Local hypoxia in the kidney also results in tubular atrophy, inflammation, and interstitial accumulation of extracellular matrix [3, 4]. Loss of vascular endothelial growth factor (VEGF) and chronic hypoxia lead to microvascular dysfunction in kidneys [3–6].

The mammalian glomerulus has a well-developed capillary tuft, and these capillaries are lined by thin fenestrated endothelial cells and podocytes that play critical coordinating roles in renal physiology as well as in innate and adaptive immunity [7, 8]. An imbalance in the decrease in endothelial repair and increase in endothelial apoptosis-after renal injury causes glomerular lesions (GLs), such as the loss of the glomerular capillaries and glomerulosclerosis [9]. In diabetic nephropathy, increased blood glucose levels cause capillary injuries [10]. Furthermore, tubulointerstitial capillaries are involved in the regulation of renal function and hemodynamics [2]. Capillary endothelial cells have a crucial function in maintaining renal homeostasis and expressing specific chemokines that control compartment-specific T-cell and monocyte recruitment during inflammation [11, 12]. This functional variability in endothelial cells is probably associated with differential susceptibilities to apoptosis and differential responses to microenvironmental changes or stimuli. Tubulointerstitial capillaries are also injured in diabetic nephropathy, resulting in a reduction in capillary density and progression of tubulointerstitial lesions (TILs) [13].

Thus, we can distinguish between the renal pathogenesis of GLs and TLs. Earlier drug-induced or spontaneous rodent models, and autoimmune disease models such as MRL/MpJ-Fas^*lpr/lpr*^ and BXSB/MpJ-*Yaa* (Yaa), in particular, have been widely used, and they manifest severe glomerulonephritis caused by immune-complex deposition [14, 15]. Male Yaa mice show more severe glomerulonephritis than females because of a Y-linked autoimmune acceleration (Yaa) mutation on the Y chromosome; however, we previously clarified that the BXSB/MpJ (BXSB)-genetic background also contributes to the progression of autoimmune disease-mediated glomerular damage [14]. In Yaa mice, GLs appeared earlier and were more severe than TILs, which appeared at a late stage in glomerulonephritis [16]. For TILs, unilateral ureteral obstruction (UUO) is usually performed to induce tubulointerstitial fibrosis in mice. UUO results in hydronephrosis, interstitial infiltration of inflammatory cells, tubular cell death from hypoxia, and collagen deposition in the tubulointerstitium, followed by the development of tubulointerstitial fibrosis [17]. A previous study identified TILs, rather than GLs, as the main cause of progressive and end stage kidney disease [18]. However, other researchers have shown the involvement of microvasculature in the advance of human and experimental animal glomerulonephritis [19–21].

Nevertheless, there are no reports clarifying the correlations between altered capillary structures and renal histopathology or renal function in GLs and TILs. The present study investigated the morphological, quantitative, and ultrastructural alterations in glomerular and tubulointerstitial capillaries using two mouse models, namely the spontaneous glomerulonephritis model and UUO model. In addition, based on an analysis of these models, we clarified the correlations between the number of local capillaries in GLs and TILs and kidney diseases severity.

## Methods

### Animals

The authors adhered to the *Guide for the Care and Use of Laboratory Animals of Hokkaido University, Graduate School of Veterinary Medicine* (approved by the Association for Assessment and Accreditation of Laboratory Animal Care International). Animal experimentation was approved by the Institutional Animal Care and Use Committee of the Graduate School of Veterinary Medicine, Hokkaido University (approval no. 13–0032, 16–0124). Experimental mice were purchased from Japan SLC Inc. (Shizuka, Japan). A maximum of five mice were held in one cage containing wood chip bedding material in a pathogen-free animal house. Food and water are provided *ad libitum* to the animals. Light and dark conditions were maintained at 1:1 ratio. Strain and number of mice used in different disease models and protocols are shown details in Additional file 1. Six month-old male Yaa mice (*N* = 12) were used for the GL model, and same-aged male BXSB mice (*N* = 12) were used as healthy controls. To create the TIL model, 8-week-old male C57BL/6 mice (*N* = 12) were subjected to UUO for 7 days. The kidney paired with the UUO kidney in the same mouse was used as a normal control. Briefly, mice were deeply anesthesized with a mixture of 0.3 mg/kg medetomidine (Kyoritsu Seiyaku, Japan), 4 mg/kg midazolam (Astellas Pharma, Japan), and 5 mg/kg butorphanol (Meiji Seika Pharma, Japan), and laparotomy in the sublumbar region was performed to ligate the right ureter tightly with silk thread at the renal hilus. Buphrenorphine hydrochloride (Otsuka Pharmaceuticals, Japan) was injected intraperitoneally at a dose rate of 0.3 mg/kg as an analgesic. Recovery from anesthesia was facilitated by intraperitoneal administration of 0.3 mg/kg atipamezole (Zenoaq, Japan).

### Sample preparation

Mice without external abnormalities were used in this experiment. The average weights of GLs and TILs model were 25.02 and 20.60 g, respectively. Urine was collected from anesthetized mice. Mice were euthanized by exsanguination from the carotid artery, and blood and kidneys were collected for serological analysis and histological examination, respectively. The kidneys were fixed with neutral buffer formalin (NBF), 4% paraformaldehyde (PFA) in 0.1 M phosphate buffer (PB, pH 7.4) or 2.5% glutaraldehyde (GTA) in 0.1 M PB.

### Serological and urinary examination

Serum levels of antibody against anti-double stranded DNA (dsDNA ab) were measured with a Mouse Anti-dsDNA Ig’s (Total A + G + M) ELISA Kit (Alpha Diagnostic International, San Antonio, TX, USA). Serum blood urea nitrogen (sBUN) and creatinine (sCr) levels in all mice were measured using a Fuji Drichem 7000v (Fujifilm, Tokyo, Japan). Urinary albumin creatinine ratios (uACR) were determined using Albuwell M and Creatinine Companion Kits (Exocell, Philadelphia, PA, USA).

### Histopathological examination

Paraffin sections of kidneys fixed with NBF or PFA were cut at a thickness of 2 μm and stained with periodic acid Schiff-hematoxylin (PAS-H) or Masson’s trichrome (MT). Immunostaining for alpha smooth muscle actin (αSMA), B220, CD3, CD34, Iba1, and interleukin 1 family, member 6 (IL-1F6/IL-36α) [22] was performed to detect myofibroblasts, B-cells, T-cells, capillary endothelial cells, macrophages, and damaged renal tubules, respectively. Staining conditions are listed in Table 1. Briefly, after deparaffinization, kidney sections were subjected to antigen retrieval. Then, slides were submerged in methanol containing 3% H_2_O_2_ for 20 min at room temperature. After blocking, sections were incubated with primary antibody overnight at 4 °C. After washing in phosphate-buffered saline (PBS), sections were incubated with secondary antibody for 30 min at room temperature, then washed and incubated with streptavidin-biotin complex (SABPO kit, Nichirei, Tokyo, Japan) for 30 min. All sections were then incubated with 3,3-diaminobenzidine tetrahydrochloride-H_2_O_2_ solution. Finally, the sections were counterstained with hematoxylin and dehydrated with an ascending series of alcohols.

**Table 1:** Summary of immunostaining conditions

	αSMA	B220	CD3	CD34	Iba1	IL-1F6/IL-36α
Antigen retrieval	CB 105 °C, 20 min	CB 105 °C, 20 min	TB 105 °C, 20 min	CB 105 °C, 20 min	0.1% pepsin 37 °C, 5 min	CB 105 °C, 20 min
Blocking	10% NGS	10% NGS	10% NGS	10% NGS	10% NGS	5% NDS
Primary antibody	Rabbit polyclonal antibodies (Abcam, Cambridge, UK) 1:3000	Rat polyclonal antibodies (Cedarlane, Ontario, Canada) 1:1000	Rabbit polyclonal antibodies (Nichirei, Tokyo, Japan) 1:200	Rabbit polyclonal antibodies (Abcam, Cambridge, UK) 1:400	Rabbit polyclonal antibodies (Wako, Tokyo, Japan) 1:2000	Goat polyclonal antibodies (R&D Systems, Minnesota, USA) 1:400
Biotinylated secondary antibody	Goat anti-rabbit (SABPO kit, Nichirei, Tokyo, Japan) 1:100	Rat anti-goat IgG (Caltag Medsystems, Buckingham, UK) 1:100	Goat anti-rabbit (SABPO kit, Nichirei, Tokyo, Japan) 1:100	Rat anti-goat IgG (Caltag Medsystems, Buckingham, UK) 1:100	Goat anti-rabbit IgG (SABPO kit, Nichirei, Tokyo, Japan) 1:100	Donkey anti-goat IgG (Santa Cruz, California, USA) 1:100

*CB* citrate buffer, *TB* tris buffer, *NGS* normal goat serum, *NDS* normal donkey serum

### Histoplanimetry

Digital images of over 30 glomeruli or over 30 tubulointerstitial areas randomly selected from each mouse were obtained at 400× magnification using an All-in-One Fluorescence Microscope BZ-X710 (Keyence, Osaka, Japan). The size and number of total cells in each glomerulus were determined using PAS-stained sections. The number of B220^+^ B-cells, CD3^+^ T-cells, Iba1^+^ macrophages, and CD34^+^ capillaries observed in the digital images of glomeruli were counted using immunohistochemical sections and a BZ-X Analyzer (Keyence). Further, glomerular damage was semi-quantitatively scored according to methods described by Ichii et al. [23]. For TILs, the numbers of B220^+^ B-cells, CD3^+^ T-cells and IL-1F6/IL-36α^+^ damaged tubules throughout the cortex were counted. Additionally, CD34^+^ capillaries, Iba1^+^ macrophages, and αSMA^+^ reaction areas in the tubulointerstitium were counted using immunohistochemical sections and a BZ-X Analyzer (Keyence), based on digital images of the renal cortex.

### Ultrastructural examination

Kidneys were pre-fixed with 2.5% GTA in 0.1 M PB for 4 h at 4 °C, post-fixed with 1% osmium tetroxide in 0.1 M PB for 2 h at 4 °C, and then dehydrated in graded alcohol and embedded in epoxy resin (Quetol 812 mixtures; Nisshin EM, Tokyo, Japan). Ultrathin sections (60 nm) were stained with uranyl acetate and lead citrate. Mounted samples were observed under a JEOL transmission electron microscope (TEM, JEM-1210; JEOL, Tokyo, Japan).

### Visualization of the renal vasculature

Vascular corrosion casts of kidney were prepared according a method described by Verli et al. [24]. Mice other than those used for the histological analysis (*N* = 4) were used for vascular casts of mice in both the control and disease groups. Briefly, after euthanasia, PBS containing EDTA, followed by a mixture of resin and catalyst (Mercox II cast, Ladd Research, Williston, USA) was perfused through left ventricles of heart. Dissected kidneys were held in a water bath at 37 °C overnight to allow polymerization with resin. Tissue surrounding the vascular bed was corroded by applying 15% potassium hydroxide for 30 h at room temperature. After washing with distilled water, casts that emerged in *t*-butyl alcohol were subjected to freeze-drying in a vacuum freeze dryer (ES-2030, Hitachi, Tokyo, Japan). The dried specimens were mounted on a specimen stub, sputter-coated using a Hitachi E-1030 ion sputter coater (Hitachi, Tokyo, Japan), and then examined by scanning electron microscopy (SEM, S-4100; Hitachi, Tokyo, Japan) with an accelerating voltage of 4 kV.

In addition, after euthanasia, rubber (Microfil, Flow Tech, Inc. Massachusetts, USA) was perfused through left ventricles according to a method described by Walker et al. [25]. Then, dissected kidneys were fixed with 4% PFA in 0.1 M PB overnight at 4 °C. Fixed kidneys were cut into 200-μm-thick sections using a microslicer (DSK Microslicer DTK-3000, Ted Pella, Inc. Redding, USA) and hydrated with an ascending series of alcohols. Finally, thick sections were cleared with methyl salicylate and examined under a BZ-X Analyzer of an All-in-One Fluorescence Microscope BZ-X710 (Keyence, Osaka, Japan) to obtain Z-stack images.

### Statistical analysis

Results are expressed as the mean ± standard error (SE). For comparisons between healthy controls and experimental mice, a nonparametric Mann–Whitney *U* test (*P* < 0.05) was utilized. The Kruskal-Wallis test was used to compare three or more populations, and multiple comparisons were performed using Scheffe’s method when significant differences were observed (*P* < 0.05). Correlations between the CD34^+^ renal capillary number and renal function or histopathological indices were analyzed using Spearman’s rank correlation coefficient (*P* < 0.05).

## Results

### Histopathological features of GL and TIL models

**Fig. 1:**
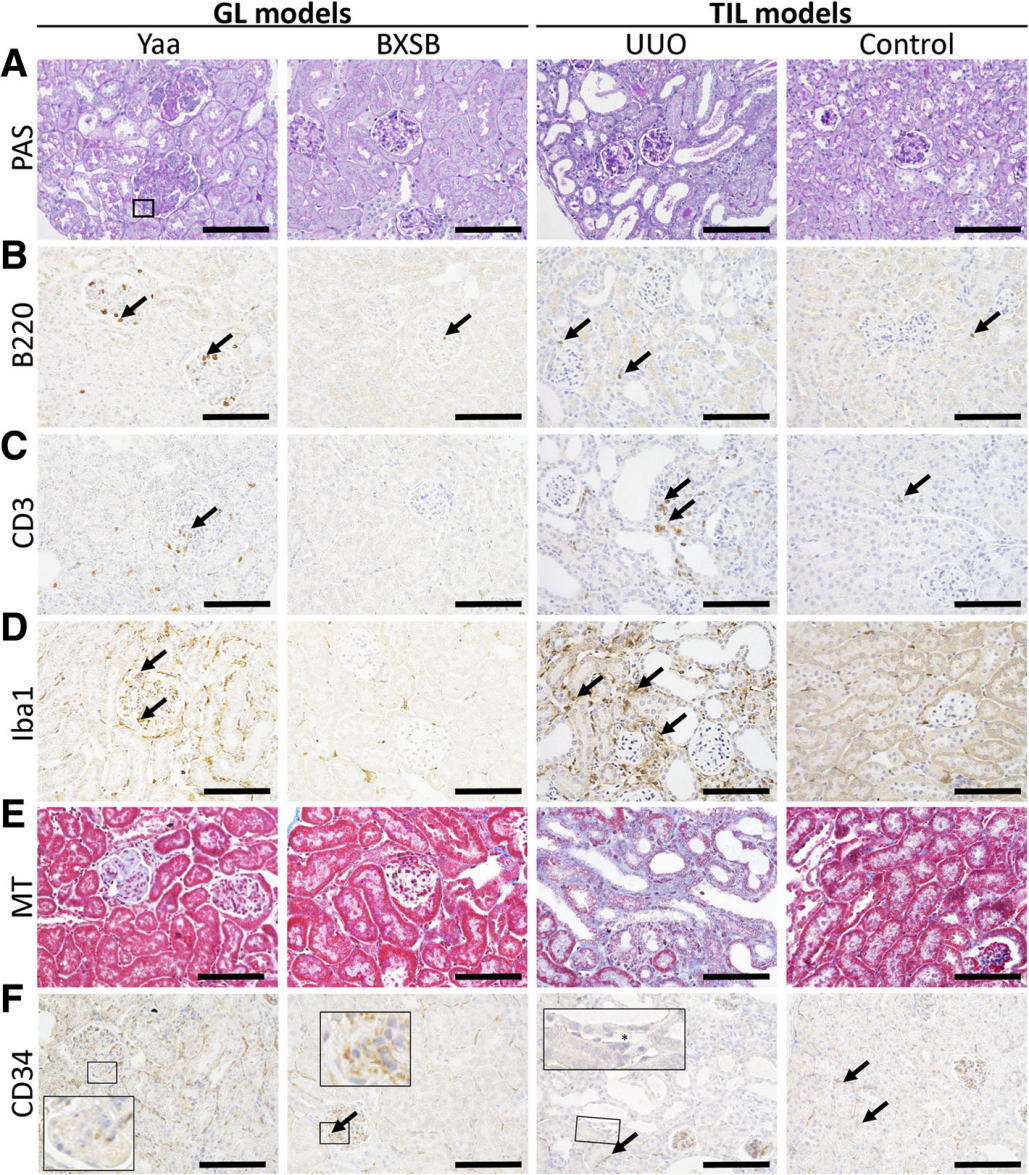
Glomerular and tubulointerstitial histopathology in glomerular lesion (GL) and tubulointerstitial lesion (TIL) mouse models. **a** Histopathological features of GLs in Yaa mice (GL model) and TILs in unilateral ureteral obstruction (UUO) mice (TIL model) and, their respective controls. Periodic acid Schiff-hematoxylin (PAS-H) staining. **b**–**d** Analysis of B220^+^ B-cell, CD3^+^ T-cells, and Iba-1^+^ macrophage infiltration in GLs of Yaa mice and TILs of UUO mice and, their respective controls. Immunohistochemistry. **e** Evaluations of renal fibrosis in the kidneys of Yaa mice and UUO mice and, their respective controls. MT staining. **f** Analysis of CD34^+^cells in GLs of Yaa mice and TILs of UUO mice and, their respective controls. Immunohistochemistry. All bars = 100 μm

In the GL model, Yaa mice clearly developed membranoproliferative glomerulonephritis features characterized by increased glomerular cell numbers and glomerular sizes, thickening of glomerular basement membranes, PAS-positive deposits, and adhesion of podocytes to parietal cells, whereas control BXSB mice did not (Fig. 1a and Additional file 2). No obvious GLs were observed in the TIL model, but UUO kidneys showed severe TILs characterized by dilated tubules with urinary casts, dilation of renal tubular lumens, and immune cell infiltrations in the tubulointerstitium (Fig. 1a). By immunohistochemistry (Fig. 1b–d), numerous B220^+^ B-cells, CD3^+^ T-cells, and Iba1^+^ macrophages were observed in the glomeruli of Yaa mice; however, few cells were found in the BXSB and TIL mice. In UUO kidneys, a few B-cells (Fig. 1b) and numerous T-cells and macrophages (Fig. 1c and d) were observed in TILs, relative to those in the control kidneys. With MT staining (Fig. 1e), aniline blue-positive sclerotic and fibrotic lesions were observed in GLs of Yaa mice and TILs of UUO kidneys, respectively, but these were not found in their respective controls.

CD34 immunostaining revealed positive reactions in the capillary endothelium of glomerulus and in the tubulointerstitium (Fig. 1f). However, in the glomeruli of Yaa mice, CD34^+^ staining was faint, although a few capillaries were observed at the peripheral regions of glomeruli. The localizations and intensities of CD34^+^ capillaries in the tubulointerstitium were comparable between Yaa and BXSB mice. However, UUO kidneys showed a decreased number and intensity of CD34^+^ reactions in TILs. Interestingly, CD34^−^ capillaries, capillaries with lumens that showed no CD34^+^ staining, were also found in UUO kidneys. CD34^+^ capillaries disappeared near damaged renal tubules with the development of fibrosis, although positive staining in the glomerulus and TILs without fibrotic features were evident. In control kidneys, CD34^+^ capillaries were observed clearly in both the glomerulus and tubulointerstitium.

### Quantitative evaluations of renal histopathological changes in GL and TIL models

**Fig. 2:**
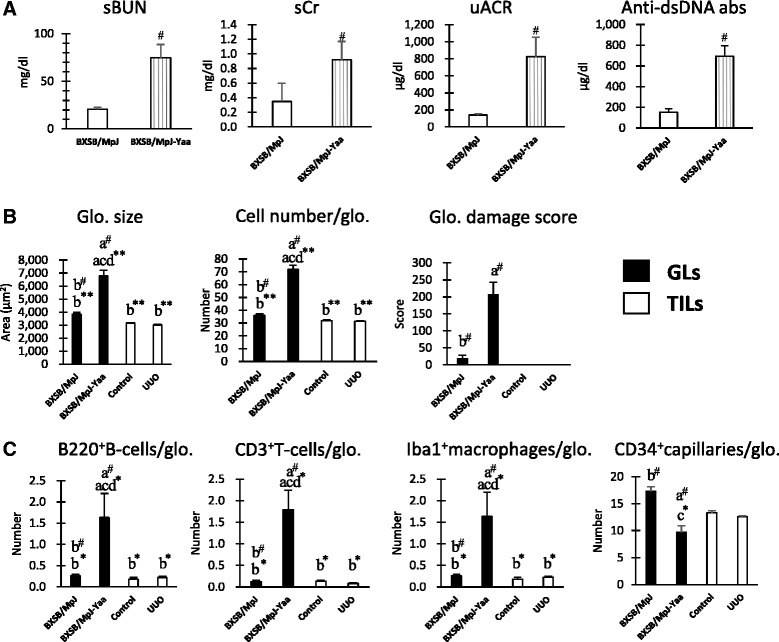
Renal function and autoimmune disease conditions in glomerular lesion (GL) models and GL-associated histological parameters in both GL and tubulointerstitial lesion (TIL) models. **a** Indices for renal function, including levels of serum blood urea nitrogen (sBUN) and serum creatinine (sCr) and urinary albumin creatinine ratios (uACRs), and for autoimmune disease conditions, namely serum levels of anti-dsDNA antibodies in Yaa mice and BXSB mice. **b** Indices for glomerular damage, namely glomerular (Glo.) size, cell number, and damage score, in model mice. **c** Indices for the number of infiltrating B220^+^B-cells, CD3^+^T-cells, and Iba1^+^macrophages and CD34^+^ capillaries in the glomeruli of model mice. Values = mean ± SE. ^#^: Significant difference from the control in the same disease group, Mann–Whitney *U* test (*p* < 0.05). ^*^ Significant difference from the other groups, Kruskal-Wallis test followed by Scheffe’s method (^*^ *p* < 0.05, ^**^ *p* < 0.01). *N* = 4. a, b, c, and d denote BXSB/MpJ, BXSB/MpJ-*Yaa,* Control, and UUO kidney, respectively

In the GL model mice, indices of renal function and autoimmune disease were determined. Yaa mice showed significantly higher levels of sBUN, sCr, uACR, and serum anti-dsDNA abs than BXSB mice (Fig. 2a).

As for GL parameters such as glomerular size, cell number, and damage score, Yaa mice showed significantly higher values than BXSB and TIL mice (Fig. 2b and Additional file 3). A similar tendency was observed in the number of B220^+^ B-cells, CD3^+^ T-cells, and Iba1^+^ macrophages per glomerulus, with Yaa mice showing higher values than BXSB and TIL mice (Fig. 2c and Additional file 3). Importantly, CD34^+^ glomerular capillaries decreased significantly in Yaa mice relative to those in BXSB mice (Fig. 2c and Additional file 3).

**Fig. 3:**
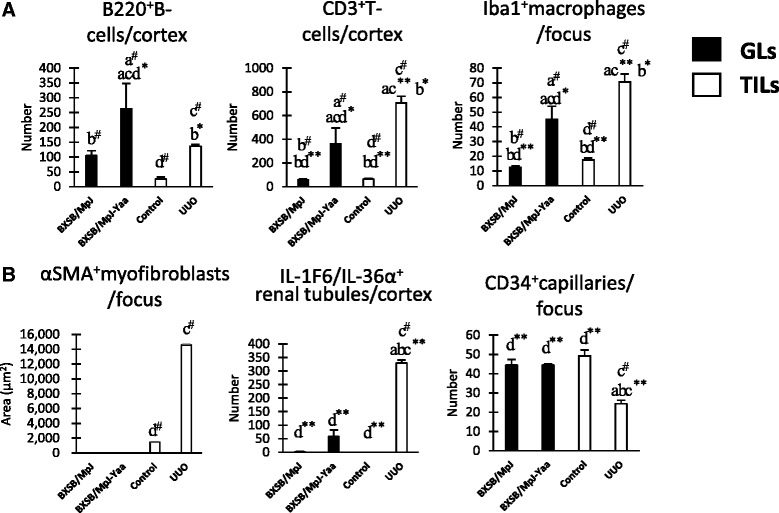
Tubulointerstitial lesion (TIL)-associated histological parameters in both glomerular lesions (GL) and TIL models. **a** Indices for the number of infiltrating B220^+^ B-cells, CD3^+^ T-cells, and Iba1^+^ macrophages in the model mice. **b** Indices for tubulointerstitial damage, including the area of αSMA^+^ myofibroblasts and the number of IL-1F6/IL-36α^+^ damaged tubules and CD34^+^ capillaries in the model mice. Values = mean ± SE. ^#^: Significant difference from the control in the same disease group, Mann–Whitney *U* test (*p* < 0.05). ^*^ Significant difference from the other groups, Kruskal-Wallis test followed by Scheffe’s method (^*^ *p* < 0.05, ^**^ *p* < 0.01). *N* = 4. a, b, c, and d denote BXSB/MpJ, BXSB/MpJ-*Yaa,* Control, and UUO kidney, respectively

As for TIL parameters such as the number of B220^+^ B-cells, CD3^+^ T-cells, Iba1^+^ macrophages, αSMA^+^ myofibroblasts, and IL-1F6/IL-36α^+^ damaged renal tubules in the renal cortex, UUO kidneys showed significantly higher values than control kidneys (Fig. 3 and Additional file 4). Yaa mice also showed significantly higher values for B220^+^ B-cells, CD3^+^ T-cells, and IL-1F6/IL-36α^+^ damaged renal tubules than BXSB mice (Fig. 3 and Additional file 4). Further, UUO kidneys showed significantly higher values for CD3^+^ T-cells, Iba1^+^ macrophages, αSMA^+^ myofibroblasts, and IL-1F6/IL-36α^+^ damaged renal tubules than kidneys in GL mice (Fig. 3 and Additional file 4). CD34^+^ tubulointerstitial capillaries decrease significantly in UUO kidneys compared to others (Fig. 3 and Additional file 4).

### Morphological alterations in capillaries of GL and TIL models

**Fig. 4:**
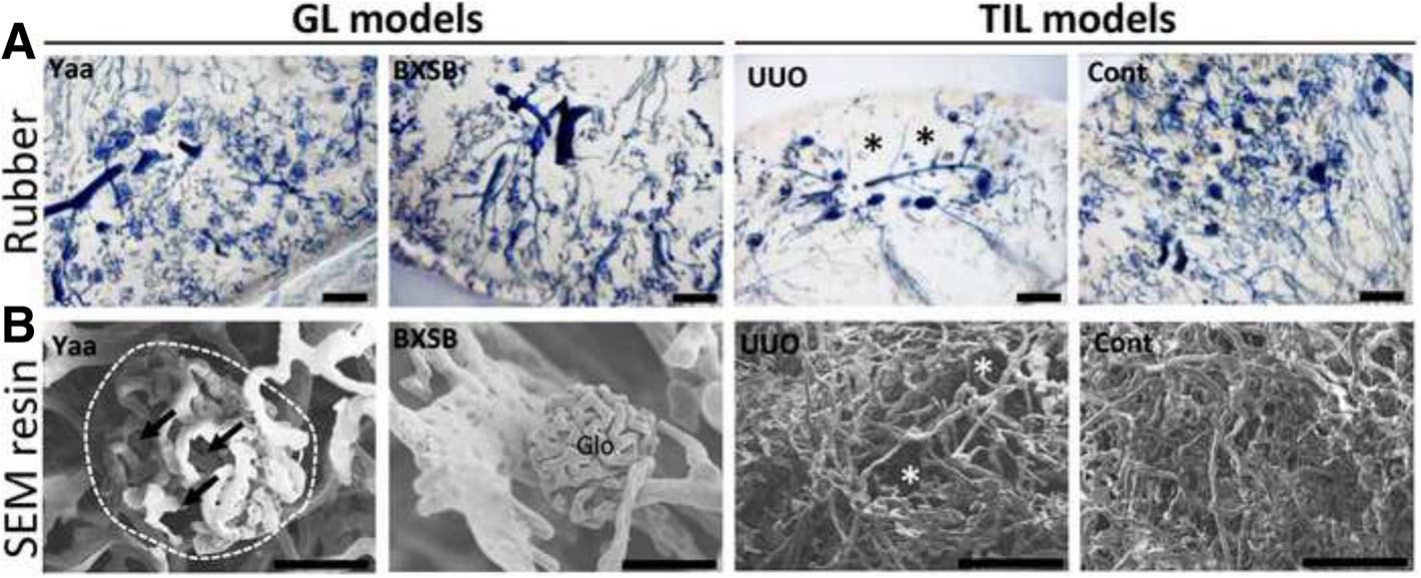
Segmental loss of capillaries in glomerular lesions (GLs) and tubulointerstitial lesions (TILs). **a** Capillary structures in the kidneys of Yaa mice, unilateral ureteral obstruction (UUO) mice, and their respective controls, visualized in thick kidney section following by rubber perfusion. Bars = 200 μm. Asterisks indicate segmental loss of tubulointerstitial capillaries. **b** Capillary structures in the kidneys of Yaa mice, UUO mice, and their respective controls, visualized by the cast method and scanning electron microscopy. Bars = 50 μm. Arrows and asterisks indicate segmental loss of glomerular and tubulointerstitial capillaries, respectively

In thick sections of rubber-injected kidneys (Fig. 4a), alterations in both glomerular and tubulointerstitial capillaries were not obvious in Yaa and BXSB mice. However, tubulointerstitial capillary loss was evident in UUO kidneys, in contrast control kidneys showed a fine capillary network. Under SEM, vascular corrosion casts (Fig. 4b) from BXSB mice showed normal tufts of glomerular capillaries, whereas Yaa mice showed segmental loss of glomerular capillaries. Interestingly, segmental loss of tubulointerstitial capillaries was observed in UUO kidneys rather than glomerular capillaries compared to those in control kidneys.

**Fig. 5:**
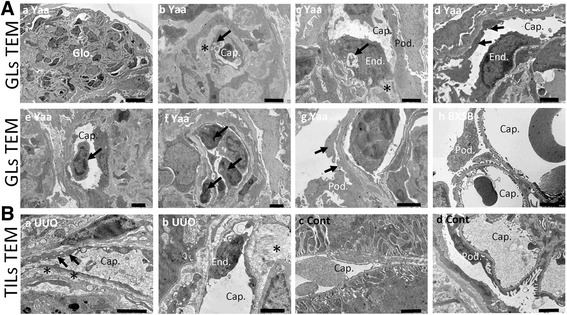
Ultrastructural alterations in glomerular and tubulointerstitial capillary morphologies in model mice. **a** Ultrastructures of glomerular capillaries in the GL model by transmission electron microscopy. Injury and loss of glomerular capillaries and podocyte injury with accumulation of increased mesangial cells and matrix in Yaa mice (panel a). Thickened endothelial cytoplasms and basement membranes (asterisk) with vacuolation (arrow) (panel b). Detaching endothelium with subendothelial vacuolation (arrow) and thickened basement membranes projecting into a capillary lumen (asterisk) (panel c). Loss of endothelial fenestration in a peripheral capillary (arrows) (panel d). Detached endothelium and immune cells in the capillary lumen (arrows) (panels e and f). Podocyte foot process effacement (arrows) (panel g). Glomerular capillaries and podocytes ultrastructures were well preserved in BXSB mice (panel h). All bars = 2 μm, except for panel a = 10 μm. **b** Ultrastructures of tubulointerstitial capillaries in the TIL model by transmission electron microscopy. Stratification of a thickened endothelial cytoplasm with vacuolation (arrows) and wide subendothelial space (asterisks) (panel a). Endothelium (arrows) detaching from a tubulointerstitial capillary with accumulation of collagen fibers around the capillary (asterisk) (panel b). Control kidneys showed normal tubulointerstitial capillaries and podocytes (panels c and d). All bars = 2 μm. Cap: capillary. End: endothelial cell. Glo: glomerulus. Pod: podocyte

In ultrastructural observation of the GL model mice by TEM, loss of glomerular capillaries, podocyte injury with accumulation of mesangial matrix, and proliferation of mesangial cells were found in glomerulosclerosed areas of glomeruli in Yaa mice (Fig. 5A-a). Glomerular capillaries showed thickened endothelial cytoplasms with vacuolation and thickened capillary basement membranes (Fig. 5A-b). Sub endothelial vacuolation was found just beneath the detaching endothelium (Fig. 5A-c). Capillary basement membranes were thickened and projected into lumens, resulting in partial occlusion of capillary lumens (Fig. 5A-c). Glomerular capillaries at the periphery showed thickened endothelial cytoplasms with loss of endothelial fenestration (Fig. 5A-d), detached endothelia, and the accumulation of immune cells in capillary lumens (Fig. 5A-e and -f). Podocyte injuries were characterized by foot process effacement in areas adjacent to thickened endothelial layers of capillaries (Fig. 5A-g). Ultrastructure were well preserved in glomerular capillaries and podocytes in BXSB mice (Fig. 5A -h).

In the TIL model, tubulointerstitial capillary injuries were clearly observed in UUO kidneys (Fig. 5B). These injuries were characterized by stratification of thickened endothelial cytoplasms with vacuolation, and wide subendothelial spaces were also found in tubulointerstitial capillaries (Fig. 5B-a). Endothelia detaching from tubulointerstitial capillaries were observed concurrently with the accumulation of collagen fibers around capillaries, resulting in narrowing of capillary lumens (Fig. 5B -b). Control kidneys showed normal glomerular and tubulointerstitial capillaries as well as podocyte morphology (Fig. 5B-c and -d).

### Correlations between the number of CD34^+^ capillaries and histopathological indices in GL and TIL models

The number of CD34^+^ glomerular capillaries in GL mice were significantly and negatively correlated with sBUN, sCr, uACR, serum levels of anti-dsDNA ab, glomerular size, glomerular cell number, glomerular damage score, and infiltrations of B220^+^ B-cells, CD3^+^ T-cells, and Iba1^+^ macrophages in glomeruli (Table 2). However, the number of CD34^+^ glomerular capillaries in TILs model mice did not correlate with glomerular histopathological parameters in these mice (Table 2). Conversely, the number of CD34^+^ tubulointerstitial capillaries was significantly and negatively correlated with the number of infiltrating B220^+^ B-cells, CD3^+^ T-cells, Iba1^+^ macrophages, IL-1F6/IL-36α^+^ damaged renal tubules, and αSMA^+^myofibroblasts in the TIL (Table 3), but only with infiltrating CD3^+^ T-cells and Iba1^+^ macrophages in GL, model (Table 3).

**Table 2:** Correlation between CD34^+^ glomerular capillaries and histopathological parameters of glomeruli in GL and TIL model mice

Parameters		Indices for glomerular histopathology						
		Glo. Size (μm^2^)	Cell number/glo.	Glo. damage score	B220^+^B-cells/Glo.	CD3^+^T-cells/Glo.	Iba1^+^macrophages/Glo.	CD34+ capillaries/Glo.
GL model	BXSB/MpJ	3850 ± 116.35^b#^	35.91 ± 1.40^b#^	18.25 ± 9.8^b#^	0.26 ± 0.03^b#^	0.12 ± 0.04^b#^	0.13 ± 0.02^b#^	17.29 ± 0.87^b#^
	BXSB/MpJ-*Yaa*	6771.94 ± 446.32^a#acd**^	71.85 ± 3.30^a#acd**^	206.25 ± 36.29^a#^	1.63 ± 0.57^a#acd*^	1.79 ± 0.45^a#acd*^	3.57 ± 0.40^a#acd*^	9.68 ± 1.25^a#ac*^
TIL model	Control kidney	3161.13 ± 0.53^b**^	31.88 ± 0.53^b**^	0	0.18 ± 0.04^b*^	0.13 ± 0.02^b*^	0.07 ± 0.01^b*^	13.3 ± 0.42^b*^
	UUO kidney	3010 ± 64.50^b**^	31.31 ± 0.47^b**^	0	0.22 ± 0.03^b*^	0.08 ± 0.01^b*^	0.03 ± 0.0^b*^	12.56 ± 0.19

Values = mean ± SE. ^#^: Significant difference from the control in the same disease group, Mann–Whitney *U* test (*p* < 0.05). ^*^Significant difference from the other groups, Kruskal-Wallis test followed by Scheffe’s method (^*^ *p* < 0.05, ^**^ *p* < 0.01). *N* = 4. Glo.: glomerular; Pod: podocyte; GLs: glomerular lesions; TILs: tubulointerstitial lesions

^a, b, c^and ^d^denotes BXSB/MpJ, BXSB/MpJ-*Yaa,* Control and UUO kidney, respectively

**Table 3:** Correlation of CD34^+^ tubulointerstitial capillaries with histopathological parameters of the tubulointerstitium in GL and TIL model mice

Parameters		Indices for tubulointerstitial histopathology					
		Ti. B220^+^B-cells/cortex	Ti. CD3^+^T-cells/cortex	Ti. Iba1^+^ macrophages/focus	Ti. αSMA^+^ myofibroblasts/focus	IL-1F6/IL-36α^+^ renal tubules/focus	CD34 + tubulointerstitial capillaries/focus
GL model	BXSB/MpJ	104.75 ± 16.70^b#^	59.75 ± 7.27^b# bd*^	12.43 ± 0.88^b# bd*^	0	1.25 ± 1.25^d**^	44.52 ± 2.76^d**^
	BXSB/MpJ-*Yaa*	263 ± 85.90^a# acd*^	362.75 ± 133.06^a# acd*^	45.25 ± 8.91^a# acd*^	0	58.5 ± 24.38^d**^	44.51 ± 0.60^d**^
TIL model	Control kidney	25.75 ± 6.90^d#^	65.5 ± 6.75^d# bd*^	17.51 ± 1.31^d# bd*^	1463.06 ± 39.12^d#^	0	49.22 ± 3.12^d**^
	UUO kidney	136 ± 6.14^c# b*^	707 ± 55.48^c# ac** b*^	70.73 ± 5.20^c# ac** b*^	14,579 ± 78.62^c#^	329.66 ± 11.01^c# abc*^	24.4 ± 1.86^c# abc*^

Values = mean ± SE. ^#^: Significant difference from the control in the same disease group, Mann–Whitney *U* test (*p* < 0.05). ^*^ Significant difference from the other groups, Kruskal-Wallis test followed by Scheffe’s method (^*^ *p* < 0.05, ^**^ *p* < 0.01). *N* = 4. Ti.: tubulointerstitial; GLs: glomerular lesions; TILs: tubulointerstitial lesions

^a, b, c^and ^d^denotes BXSB/MpJ, BXSB/MpJ-*Yaa,* Control and UUO kidney, respectively

## Discussion

For the GL model using Yaa mice, the number of glomeruli with cells positive for CD34, a representative marker of endothelial cells, significantly and negatively correlated with serum levels of autoantibody and deterioration in renal function, as well as increased numbers of infiltrating cells in the glomeruli. TILs in GL models showed an increase in cell infiltration and damaged renal tubules, but not myofibroblasts; however, the number of CD34^+^ capillaries in TILs was comparable between BXSB and Yaa mice and did not correlate with any renal pathological parameters. Further, no significant GLs were noted in UUO models. The Yaa mouse is a model of spontaneous autoimmune-mediated membranoproliferative glomerulonephritis, with hyperproliferation of pathogenic B-cells resulting from a Yaa mutation that contributes to autoantibody production, activation of pathogenic T-cells, and secretion of pro-inflammatory cytokines that contribute to the development of GLs [16, 22, 26–28]. Therefore, GL events appear earlier and were more severe than TILs in Yaa mice, whereas severe TILs, with fibrotic features characterized by an increase in myofibroblasts, were prominent end stage kidney disease in Yaa mice. However, because glomerular efferent arterioles directly connect to branches of peritubular/tubulointerstitial capillaries, a reduction in blood supply to the glomerulus because of GLs might affect TILs. Collectively, the study data indicate that a decrease in CD34^+^ cells in glomerular capillaries correlates with the progression of GLs in autoimmune disease-prone Yaa mice.

In GL models, glomerular capillaries had narrow lumens, thickened endothelial cytoplasms with vacuolation, loss of endothelial fenestration, and detached luminal endothelia in capillary lumens. Although renal tubulointerstitial capillary injury, particularly capillary basement membrane multilamination, is prominent feature in chronic microvascular injury in renal allograft rejection [29, 30], we did not find such features in the kidneys of autimmene-disease prone Yaa mice. These results indicate that CD34^+^ capillaries decreased with endothelial cell injury, evident by their morphological changes. These capillary endothelial cells seemed to play crucial roles in the progression of glomerulonephritis and renal dysfunction. Briefly, several studies have shown an imbalance in endothelial cell proliferation and death that is associated with a decrease in renal capillaries leads to the progression of kidney disease [19, 20, 31–33]. Importantly, hypoxia promotes GLs, similar to mesangial cell proliferation in patients with lupus nephritis and in MRL/MpJ-Fas^*lpr/lpr*^ mice [34]. Therefore, the loss of CD34^+^ glomerular capillaries and their morphological changes in Yaa mice occur after the progression of GLs, including the development of membranoproliferative lesions and inflammation.

Furthermore, podocytes are involved in the maintenance of healthy intracapillary environments through crosstalk with glomerular endothelial cells and as a source of VEGF in glomeruli [35, 36]. Podocyte injury is a critical event that causes albumin hyperfiltration from glomerular capillaries [37]. Indeed, our ultrastructural study showed podocyte foot process effacement in GL model mouse kidneys, but not in those of the TIL model mouse. Thus, pathological changes in the glomerular microenvironment resulting from injury of podocytes and capillary endothelia coordinately aggravate GLs and lead to an elevation in uACR.

Capillaries in the tubulointerstitium are essential for renal oxygen supply and maintenance of kidney tubulointerstitial hemodynamics [38, 39]. TILs activate the endothelium, which may correlate with enhanced inflammation and activation of coagulation that favors further capillary and interstitial injury [40, 41]. Eventually, persistent TILs cause a loss of capillaries in the tubulointerstitium [42]. In the present study, TIL models were created by UUO. The advantage of UUO was that diseased and control kidneys could be obtained from the same mice. TILs model mice clearly showed a decrease in CD34^+^ capillaries in the tubulointerstitium with the progression of TILs, characterized by an increase in infiltrating cells and myofibroblasts, as well as damaged tubules in the tubulointerstitium. Furthermore, the ultrastructural study revealed capillary injuries in TILs in detail. These injuries were characterized by thickened and stratified endothelial cytoplasms with vacuolation, loss of fenestration, detaching endothelia with subendothelial vacuolation, and accumulation of collagen fibers beneath the capillary basement membrane. Thus, our results clearly indicate pathological correlations between TILs and capillary injury and/or loss in tubulointerstitium in UUO-based TIL models.

In particular, the number of infiltrating CD3^+^ cells strongly correlated with the number of CD34^+^ capillaries in TILs of UUO models. Therefore, we presumed that interstitial T-cells mediate inflammation and the accumulation of macrophages in the tubulointerstitium. Importantly, an increase in Iba-1^+^macrophages significantly correlated with a decrease in CD34^+^ capillaries in the present study. Another study showed that apoptosis of endothelial cells triggered capillary regression by blocking blood flow to the site of apoptosis in macrophage-dependent cell death [43]. Renal fibrosis results from reduced endothelial proliferation following alterations in local expression of both angiogenic and antiangiogenic factors, and this imbalance is mediated by macrophage-associated cytokines, such as interleukin 1 beta, and vasoactive mediators [38]. Based on these findings, we considered tubulointerstitial inflammation, especially macrophage infiltration, to underlie injury of capillary endothelial cells and the subsequent net loss of capillaries.

The present study revealed a significant correlation between the number of CD34^+^ capillaries and the numbers of IL-1F6/IL-36α^+^ damaged renal tubules and αSMA^+^ myofibroblasts. Evidently, loss of tubulointerstitial capillaries caused TILs because of local hypoxia, because tubulointerstitial capillaries and renal tubules show functional crosstalk to maintain normal renal interstitial structure and function, including preserving the blood supply to maintain tubular epithelial cells. This speculation is strongly supported by previous study indicating that renal ischemia caused by vascular obliteration is a major contributor to renal fibrosis [3]. Moreover, the tubular epithelium is a source of VEGF in the tubulointerstitium [44]. Therefore, we concluded that injury and/or loss of tubulointerstitial capillaries contribute to the progression of TILs, and fibrosis and tubular damage in particular, in UUO kidneys.

In this study, we examined the decrease in CD34^+^ renal capillaries associated with the progression of kidney disease using murine GLs and TILs models. The quantitative changes in CD34^+^ capillaries could represent two possible pathological events: 1) a decrease in capillary number, or 2) a decrease in CD34^+^ cell number. As shown in the figures, the numbers of local capillaries decreased in GLs and TILs. However, interestingly, CD34^−^capillaries were found in TILs of UUO models, but not in GLs of Yaa mice. CD34 is a single-pass transmembrane sialomucin protein that is expressed on hematopoietic stem cells and in vascular-associated tissue [45]. It is an important adhesion molecule required for lymphocytes to enter lymph nodes [46]. Kusano et al. suggested that the loss of CD34^+^ capillaries was a result of the disappearance of cell markers in phenotypically altered endothelium [21]. Therefore, a decrease in CD34^+^ cells in kidneys indicates a decrease in renal capillaries, as well as altered functions of endothelial cells resulting from decreased CD34 due to capillary injury. In a future study, we will compare the loss of renal capillaries and decreased expression of CD34 as contributors to kidney disease progression. Results of this study indicate the importance of morphology and density of local capillaries in human and animal kidney diseases. Blood vessel has been caught the attention recently as it contribute to stem cell niches in various organs, signifying that vascular system may serve a preserved concerned role for stem cells throughout the body. Moreover, the kidney is a highly vascularized organ, and this study shows that GLs and TILs are associated with reduced number of glomerular capillaries and tubulointerstitial capillaries, respectively. Specially, there is a strong correlations between the number of local capillaries in GLs and TILs and the severity of kidney diseases. Pathological changes might also affect vasculature-associated stem cell niche in the kidney. In the future, we will investigate these possibilities.

## Conclusions

Injury and/or loss of the local renal microvasculature is a prominent feature of kidney disease. Defective microvasculature results from profound infiltration of inflammatory cells and cross-talk with surrounding tissues. The progression of local capillary damage can be considered a double-edged sword, i.e., both the cause and the effect of kidney disease. Therefore, we conclude that injury and the subsequent loss of local capillaries contributes to the progression of lesions in respective area and kidney dysfunction.

## Additional files

Additional file 1: Outline of the study: Strain and number of mice used in different disease models and protocols. (PDF 24 kb)

Additional file 2: Glomerular lesion model mice show thickening of glomerular basement membranes (GBMs). (PDF 131 kb)

Additional file 3: Glomerular histopathology indices in glomerular lesion and tubulointerstitial lesion models. (PDF 18 kb)

Additional file 4: Tubulointerstitial histopathology indices in glomerular lesion and tubulointerstitial lesion models. (PDF 19 kb)

## Abbreviations

ds-DNA: Double stranded-DNA
g: Gram
GLs: Glomerular lesions
GTA: Glutaraldehyde
IL-1F6: Interleukin 1 family, member 6
NBF: Neutral buffer formalin
PAS-H: Periodic acid-Schiff-hematoxylin
PB: Phosphate buffer
PBS: Phosphate-buffered saline
PFA: Paraformaldehyde
sBUN: Serum blood urea nitrogen
sCr: Serum creatinine
SE: Standard error
TILs: Tubulointerstitial lesions
uACR: Urinary albumin creatinine ratio
UUO: Unilateral ureteral obstruction
VEGF: Vascular endothelial growth factor
αSMA: Alpha smooth muscle actin
