# Supplementary material for: Local CD34-positive capillaries decrease in mouse models of kidney disease associating with the severity of glomerular and tubulointerstitial lesions
Source: BMC Nephrol. 2017 Sep 4;18:280. doi: 10.1186/s12882-017-0694-3 (PMC5584339; doi:10.1186/s12882-017-0694-3)
Supplement: Supplementary file 1 — Outline of the study: Strain and number of mice used in different disease models and protocols. (PDF 24 kb) [file 12882_2017_694_MOESM1_ESM.pdf]

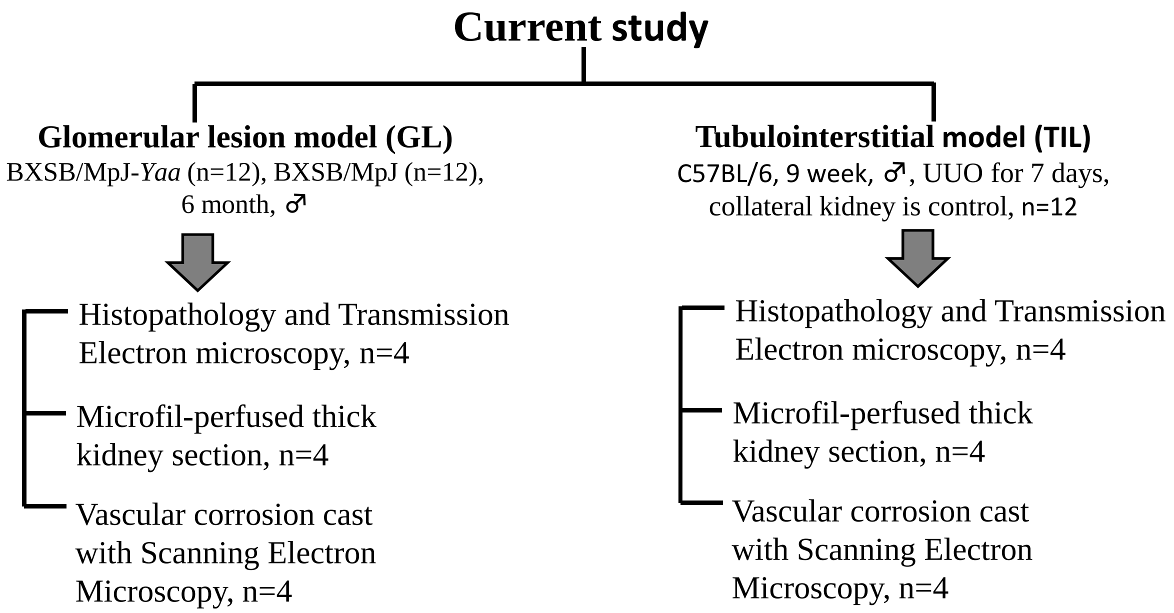

Additional file 1: Summary of the current study. Six-month-old male BXSB/MpJ-*Yaa* mice were used for the glomerular lesion model, and male BXSB/MpJ mice at the same age were used as the controls for this model. Unilateral ureteral obstructed kidneys of 9-week-old C57BL/6 mice were used for the tubulointerstitial model; paired kidneys from the same animals were used as controls. Mice belong to GL and TIL model were subjected for routine histopathology, Transmission Electron Microscopy, Microfil perfusion and Scanning Electron Microscopy. N = number of mice, UUO: unilateral ureteral obstruction.
