# Supplementary material for: Local CD34-positive capillaries decrease in mouse models of kidney disease associating with the severity of glomerular and tubulointerstitial lesions
Source: BMC Nephrol. 2017 Sep 4;18:280. doi: 10.1186/s12882-017-0694-3 (PMC5584339; doi:10.1186/s12882-017-0694-3)
Supplement: Supplementary file 2 — Glomerular lesion model mice show thickening of glomerular basement membranes (GBMs). (PDF 131 kb) [file 12882_2017_694_MOESM2_ESM.pdf]

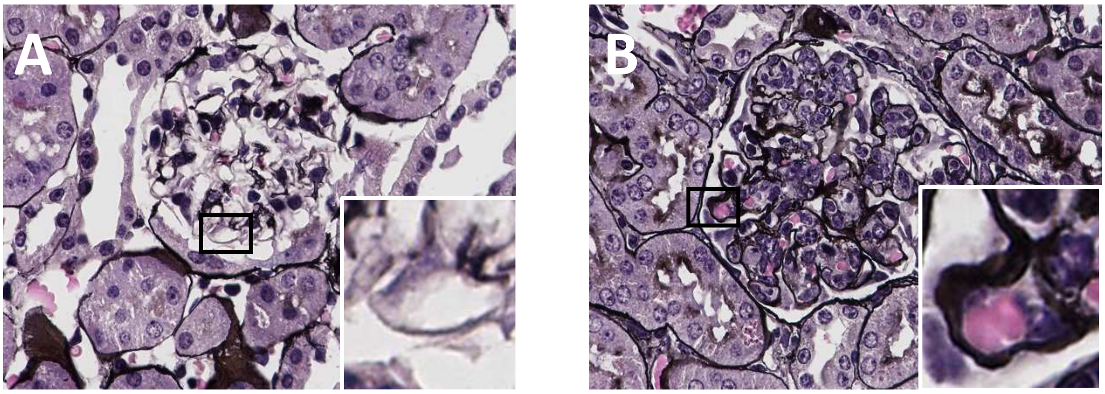

Additional file 2: Thickening of glomerular basement membranes (GBMs) in Glomerular lesion model mice .

A. BXSB/MpJ mice show normal glomerular basement membranes.

B. BXSB/MpJ-Yaa shows thickening of GBMs by periodic acid methanamine silver (PAM) staining. All bars = 100 µm.
