# Supplementary material for: Local CD34-positive capillaries decrease in mouse models of kidney disease associating with the severity of glomerular and tubulointerstitial lesions
Source: BMC Nephrol. 2017 Sep 4;18:280. doi: 10.1186/s12882-017-0694-3 (PMC5584339; doi:10.1186/s12882-017-0694-3)
Supplement: Supplementary file 3 — Glomerular histopathology indices in glomerular lesion and tubulointerstitial lesion models. (PDF 18 kb) [file 12882_2017_694_MOESM3_ESM.pdf]

Additional file 3: Quantitative evaluation of indices for glomerular histopathology in GL and TIL model mice.

**Parameters**		**Indices for glomerular histopathology**						
		**Glo. Size (μm^2)**	**Cell number/glo.**	**Glo. damage score**	**$B220^+$B-cells/ Glo.**	**$CD3^+$T-cells/Glo.**	**$Iba1^+$macrophages /Glo.**	**CD34+ capillaries/Glo.**
**GL model**	BXSB/MpJ	3850±116.35 $^{b\#}$	35.91±1.4 $^{b\#}$	18.25±9.8 $^{b\#}$	0.26±0.03 $^{b\#}$	0.12±0.04 $^{b\#}$	0.13±0.02 $^{b\#}$	17.29±0.87 $^{b\#}$
	BXSB/MpJ-*Yaa*	6771.94±446.32 $^{a\# acd**}$	71.85±3.3 $^{a\# acd**}$	206.25±36.29 $^{a\#}$	1.63±0.57 $^{a\# acd*}$	1.79±0.45 $^{a\# acd*}$	3.57±0.4 $^{a\# acd*}$	9.68±1.25 $^{a\# ac*}$
**TIL model**	Control kidney	3161.13±0.53 ^{b**}	31.88±0.53 ^{b**}	0	0.18±0.04 ^{b*}	0.13±0.02 ^{b*}	0.07±0.01 ^{b*}	13.3±0.42 ^{b*}
	UUO kidney	3010±64.5 ^{b**}	31.31±0.47 ^{b**}	0	0.22±0.03 ^{b*}	0.08±0.01 ^{b*}	0.03±0.0 ^{b*}	12.56±0.19

Values = mean ± SE. $^{\#}$: Significant difference from the control in the same disease group, Mann-Whitney *U* test ($p < 0.05$). * Significant difference from the other groups, Kruskal-Wallis test followed by Scheffe's method ($^{*}p < 0.05$, $^{**}p < 0.01$). N = 4. Glo.: glomerular; Pod: podocyte; GLs: glomerular lesions; TILs: tubulointerstitial lesions. a, b, c and d denotes BXSB/MpJ, BXSB/MpJ-*Yaa*, Control and UUO kidney, respectively.
