# Supplementary material for: Local CD34-positive capillaries decrease in mouse models of kidney disease associating with the severity of glomerular and tubulointerstitial lesions
Source: BMC Nephrol. 2017 Sep 4;18:280. doi: 10.1186/s12882-017-0694-3 (PMC5584339; doi:10.1186/s12882-017-0694-3)
Supplement: Supplementary file 4 — Tubulointerstitial histopathology indices in glomerular lesion and tubulointerstitial lesion models. (PDF 19 kb) [file 12882_2017_694_MOESM4_ESM.pdf]

Additional file 4: Quantitative evaluation of indices for tubulointerstitial histopathology in GL and TIL model mice.

Parameters		Indices for tubulointerstitial histopathology					
		**Ti. B220$^+$B-cells/ cortex**	**Ti. CD3$^+$T-cells/ cortex**	**Ti. Iba1$^+$ macrophages/focus**	**Ti. αSMA$^+$ myofibroblasts/ focus**	**IL-1F6/IL-36α$^+$ renal tubules/focus**	**CD34+tubulointerstitial capillaries/focus**
**GL model**	BXSB/MpJ	104.75±16.7 $^{b\#}$	59.75±7.27 $^{b\# \; bd*}$	12.43±0.88 $^{b\# \; bd*}$	0	1.25±1.25 ^{d**}	44.52±2.76 ^{d**}
	BXSB/MpJ-*Yaa*	263±85.9 $^{a\# \; acd*}$	362.75±133.06 $^{a\# \; acd*}$	45.25±8.91 $^{a\# \; acd*}$	0	58.5±24.38 ^{d**}	44.51±0.6 ^{d**}
**TIL model**	Control kidney	25.75±6.9 $^{d\#}$	65.5±6.75 $^{d\# \; bd*}$	17.51±1.31 $^{d\# \; bd*}$	1463.06±39.12 $^{d\#}$	0	49.22±3.12 ^{d**}
	UUO kidney	136±6.14 $^{c\# \; b*}$	707±55.48 $^{c\# \; ac** b*}$	70.73±5.2 $^{c\# \; ac** b*}$	14579±78.62 $^{c\#}$	329.66±11.01 $^{c\# \; abc*}$	24.4±1.86 $^{c\# \; abc*}$

Values = mean ± SE. $^{\#}$: Significant difference from the control in the same disease group, Mann-Whitney *U* test ($p < 0.05$). * Significant difference from the other groups, Kruskal-Wallis test followed by Scheffe's method ($^{*}p < 0.05$, $^{**}p < 0.01$). N = 4. Ti.: tubulointerstitial; GLs: glomerular lesions; TILs: tubulointerstitial lesions. a, b, c and d denotes BXSB/MpJ, BXSB/MpJ-*Yaa*, Control and UUO kidney, respectively.
